# Self-reported oral health and oral health-related quality of life among older adults receiving home care services in South-eastern Norway

**DOI:** 10.2340/aos.v84.43425

**Published:** 2025-04-09

**Authors:** Hero Ibrahim Hassan, Marte-Mari Uhlen-Strand, Vibeke Ansteinsson, Ragnhild Hellesø, Ewa A. Sz. Hovden, Rasa Skudutyte-Rysstad

**Affiliations:** a Department of Public Health Science, Faculty of Medicine, University of Oslo, Oslo, Norway; b Oral Health Centre of Expertise in Eastern Norway, Oslo, Norway

**Keywords:** Older adults, self-reported oral health status, oral health-related quality of life, subjective dry mouth, tooth loss, pain and discomfort

## Abstract

**Objective:**

To map self-reported oral health status, oral health-related quality of life (OHRQoL), and associated factors among older adults receiving home care services (HCS) in south-eastern Norway.

**Material and methods:**

For this cross-sectional study, older adult users of HCS, aged ≥ 65 years were interviewed using an interviewer-administered questionnaire. Information about demographics, number of medicines, and Activity of Daily Living (ADL-score) was obtained. Self-reported oral health status and subjective dry mouth (Summated Xerostomia Inventory-Duch version, SXI-D) were assessed. OHRQoL was measured using Oral Health Impact Profile-14 (OHIP-14).

**Results:**

Of 116 participants (mean age 83 years), 52.6% were female. Half of the participants reported missing < 5 of their natural teeth and 6.9% were edentulous. Pain and discomfort were reported by 16.4%, and subjective dry mouth was common (40.7%). Poor oral health status was reported by 8.6%, and experiencing problems or discomfort fairly often or very often was reported by 20.7%. Good self-perceived oral health was reported by 61%. Younger (< 75 years) and less dependent (ADL < 2) individuals and those missing ≥ 5 natural teeth reported a negative impact on OHRQoL more often.

**Conclusions:**

Half of older adults receiving HCS in Norway retain most of their natural teeth, few are edentulous and xerostomia is common. Younger and less dependent individuals and those missing ≥ 5 natural teeth reported a negative impact on OHRQoL more often.

## Introduction

The proportion of older adults is increasing rapidly, and by 2050, 16% of the global population is expected to be 65 years or older [1, 2] Because of improvements in general health, life expectancy is increasing. In addition, more people are able to retain their natural teeth for life [3, 4]. However, challenges of aging, such as cognitive impairment and decreased muscle strength and coordination, cause many older adults to become dependent on help, and present difficulties regarding oral health care [5].

In Norway, care for dependent older adults is governed by national legislation, which enables many care-dependent older adults to live at home with supervision and support provided by home care services (HCS) [6]. Norwegian HCS are organized to allow individuals with special assistance needs due to illness or disabilities to apply for HCS where they live or have temporary residency [6]. As users of HCS, older adults are entitled to free dental treatment in the public dental health service (PDS) [7]. However, according to Statistics Norway, only 20.7% of the care-dependent older adults in HCS who are entitled to dental care from PDS utilize their services [8].

Due to significant improvements in oral health in Norway in recent decades, many older adults now retain their natural teeth, and few are edentulous [9]. International studies have reported that care-dependent older adults have poor oral health status, with fewer natural teeth than healthy older adults in the same age group [10]. Moreover, care-dependent older adults in HCS have been shown to have inferior oral health status compared with institutionalized individuals [11]. However, previous studies on oral health among dependent older adults have mainly focused on institutionalized older adults [12], and data on self-reported oral health status for older adults in HCS in Norway are limited.

The World Health Organization (WHO) defines oral health as a key indicator of overall health, well-being, and quality of life [13]. Oral health is based on adequate oral function, and the absence of diseases and pain or discomfort [14]. Dental caries, tooth loss, xerostomia, and mucosal lesions represent a high burden of oral health problems and may affect daily life performance in older adults [15]. Traditionally, oral health has been determined by findings from the clinical and radiographic examinations. However, subjective oral health indicators are important to provide information about how oral conditions affect the daily life of individuals [16]. The impact of oral health on quality of life is conceptualized as oral health-related quality of life (OHRQoL) [17]. Although oral health is known to affect the quality of life [1, 18], little is known about OHRQoL among care-dependent older adults living at home and receiving help from HCS [19].

This study aimed to map self-reported oral health status, OHRQoL among older adults receiving assistance from HCS in south-eastern Norway, and to explore the impact of associated factors: self-reported oral health, background and general health variables on OHRQoL.

## Materials and methods

### Participants and setting

The participants in this study were older adult users of HCS from four different municipalities from both urban (Oslo and Drammen) and rural (Hamar and Askim) areas in south-eastern Norway. Inclusion criteria were age ≥ 65 years, being a user of HCS, and being cognitively and physically able to be interviewed. Exclusion criteria were not being competent to consent and with a limited knowledge of Norwegian. The participants were recruited by HCS personnel in connection with home visits. All users of HCS who met the inclusion criteria were asked if they wanted to participate in the study. Older adults who consented to participate in the study were contacted by the research group by telephone to arrange a time for an interview at their home. In each municipality, two members of the research team consisting of dentists (*n* = 2), dental hygienists (*n* = 4), and researchers (*n* = 2) visited the participants and interviewed them using an interviewer-administered questionnaire, asking questions verbally and typing the answers on an iPad. The duration of interviews varied from 20 to 60 min.

Data collection was performed from December 2020 to January 2023. Due to the coronavirus disease 2019 (COVID-19) pandemic restrictions, data collection was interrupted several times and took longer than planned.

### Questionnaire

The questionnaire consisted of participants’ background information, their general health, and self-reported oral health variables. The questionnaire was pretested on two volunteers aged > 70 years living at home, who were not users of HCS and did not participate in the study. After minor linguistic and structural adjustments based on the pretest, the questionnaire was ready for use.

Background variables collected included sex, age, living situation, level of education, and household income. For statistical analyses, the study population was categorized into the following age groups: 65–74, 75–84, and ≥ 85 years. The living situation was assessed by asking if the participant lived alone (Yes/No). Household income was registered based on yearly income (before tax) and recorded as low (≤ 300,000 NOK), middle (301–450,000 NOK), and high (> 450,000 NOK). Participants’ highest completed education was collected and categorized as basic (primary school or lower), middle (secondary school/vocational training), and higher (college/university level).

Information about the number of medicines and Activities of Daily Living (ADL) scores were obtained from the patient journal system Gerica used by the HCS. Information on the number of medicines used was categorized as 0–4, 5–10, and > 10 medicines. ADL scores were dichotomized according to Norwegian national guidelines into scores ≤ 2 and > 2 [20]. A score > 2 means that the person has some difficulty with or is dependent on help from others in managing activities in their daily life.

Questions about self-reported oral health included: number of teeth (no missing teeth, missing 1– 4 teeth, missing 5–10 teeth, missing > 10 teeth, and having no natural teeth left); tooth replacements (crown, bridge, implant, full or partial denture), with the response alternatives Yes/No/I don’t know; and oral symptoms in the last month (bleeding gingiva, loose teeth, bad taste or bad breath, oral pain or discomfort), with response alternatives Yes/No. Self-perceived oral health status was assessed with one single question: ‘How do you feel about your oral health status?’ with three response alternatives (good, average and poor).

The Summated Xerostomia Inventory–Dutch version (SXI-D) was used for mapping the severity of experienced dry mouth symptoms [21]. The SXI-D is a validated self-reported questionnaire with five statements [21, 22], and three response alternatives (never = 1, sometimes = 2, often = 3). SXI-D sum scores range from 5 to 15 points with a higher sum score indicating more severe of experienced of problems related to dry mouth.

OHRQoL was measured using the shortened Norwegian version of the Oral Health Impact Profile-14 (OHIP-14) instrument. The OHIP-14 questionnaire consists of 14 items related to oral health in the last 12 months, divided into seven dimensions: functional limitation, physical pain, psychological discomfort, physical disability, psychological disability, social disability, and handicap [23]. The responses are rated on a 5-point Likert scale (0 = never, 1 = hardly ever, 2 = occasionally, 3 = fairly often, and 4 = very often) with sum scores ranging from 0 to 56 and a higher sum score indicating poorer OHRQoL. For the analyses, the answers were dichotomized into never/hardly ever/occasionally = 0 and fairly often/very often = 1. Responding fairly often/very often to one or more questions on OHIP-14 indicated a negative impact on OHRQoL.

### Ethics

Ethical approval for this study was obtained from the Regional Ethics Committee (REK 2020/32692) and the Norwegian Agency for Shared Services in Education and Research (Sikt, 540095). All participants gave their written informed consent. The study is reported in accordance with the guidelines provided by ‘Strengthening the Reporting of Observational Studies in Epidemiology’ (STROBE) [24].

### Statistical analysis

Nettskjema software developed and operated by the University of Oslo (UiO) was used for designing the electronic questionnaire and for data collection. The platform Services for Sensitive Data (TSD) at UiO was used to store the data. Frequency and percentage distributions were used to describe the data. The Chi-square and Fisher exact test were used to explore possible associations between categorical variables. Because the continuous variables did not follow a normal distribution, Kruskal–Wallis and Mann–Whitney *U* tests were used to detect differences in median values. All analyses were performed using Stata SE version 17 (Stata Corp. LLC version 17, Texas, USA), and the statistical significance level was set at α = 0.05.

## Results

Of the 116 older adults who were interviewed, 61 (52.6%) were female, and the mean age was 83 years (SD 8.89, range 65–102 years). Two-thirds (67.3%) of the respondents had middle or higher education (Table 1). Thirty-six percent stated that they had a low income, and the majority lived alone. Seventy percent used ≥ 5 medications and most participants had an ADL sum score > 2, indicating a high level of dependency. The distribution of participants in relation to background and general health variables is presented in Table 1.

**Table 1:** Distribution of participants according to background and general health variables (*N* = 116).

Background and general health variables	*N*	%
Sex		
Female	61	52.6
Male	55	47.4
Age		
65–74	23	19.8
75–84	33	28.5
≥ 85	60	51.7
Living alone		
Yes	91	78.5
No	25	21.5
Education		
Basic	33	28.4
Middle	51	44.0
Higher	27	23.3
Unknown	5	4.3
Household income		
Low	42	36.2
Middle	27	23.3
High	21	18.1
Unknown	26	22.4
Number of medicines		
0–4	28	24.1
5–10	56	48.3
> 10	26	22.4
Unknown	6	5.2
ADL		
Score ≤ 2	28	24.1
Score > 2	85	73.3
Unknown	3	2.6
ADL mean score (SD)	2.5 (0.77)	

ADL: Activity of Daily Living.

### Self-reported oral health variables

Table 2 shows that 50% of the older adults in the study reported to be missing < 5 of their natural teeth. Fixed prosthetic appliances were common, half of the participants reported having crowns and one-third of bridges. Almost one in five reported pain or discomfort during the last month. Good self-perceived oral health was reported by 61%.

**Table 2:** Distribution of participants according to self-reported oral health status and symptoms (*N* = 116).

Self-reported oral health status and symptoms	*N*	%
Number of teeth		
Dentate, have all natural teeth	14	12.0
Missing 1–4 teeth	44	38.0
Missing 5–10 teeth	29	25.0
Missing > 10 teeth	21	18.1
Edentulous	8	6.9
Tooth replacements		
Crown	59	50.8
Bridge	38	32.8
Tooth implant	8	6.9
Partial denture	14	12.1
Full denture	18	15.5
Oral symptoms last month		
Pain/discomfort	19	16.4
Bleeding gingiva	17	14.7
Loose teeth	11	9.5
Bad taste or bad breath	13	11.2
Self-perceived oral health		
Good	71	61.2
Average	35	30.2
Poor	10	8.6

### Subjective dry mouth

**Figure 1:**
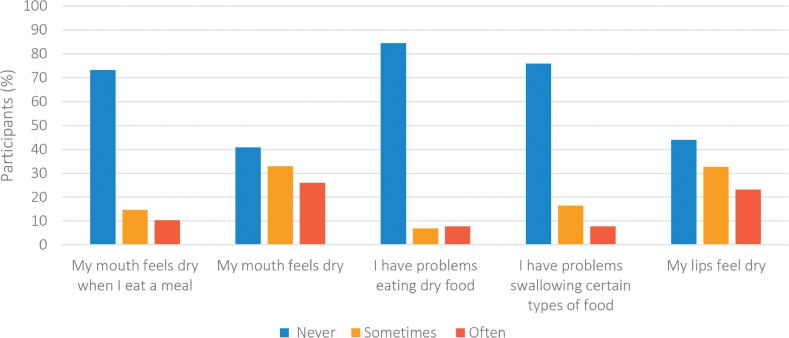
Frequency distribution of participants in relation to reported experiences of subjective dry mouth (*N* = 116).

Figure 1 shows the frequency distribution of participants in relation to reported experience of subjective dry mouth. The overall prevalence of dry mouth symptoms (answered ‘often’ to one or more questions of SXI-D) was 40.7% and the median SXI-D score was 7 with an interquartile range (IQR) of 6–9. The most frequently reported dry mouth symptoms were ‘my mouth feels dry’ followed by ‘my lips feel dry’. One in four reported problems with swallowing certain types of food and that their mouth felt dry when they ate a meal. There were no statistically significant differences between SXI-D median scores and medication use.

### Oral health-related quality of life

One in five older adults in the study reported having experienced problems or discomfort fairly often/very often, whereas no problems or discomfort at all were reported by 44.7%. The median OHIP-14 score in the study population was 1 (IQR 0–4). The proportion of participants reporting problems or discomfort within the dimensions varied from 2.6% to 11.2%. Functional limitation was reported most often (11.2%), followed by physical pain (9.5%) and psychological discomfort (8.6%). Psychological disability and handicap were reported by 6.9% and 6.0%, respectively. The most prevalent problems reported by participants were problems in pronouncing words and being self-conscious followed by a worsened sense of taste, painful aching in the mouth, discomfort eating food and life to be less satisfying (Table 3).

**Table 3:** Distribution of respondents in relation to dimensions and items of Oral Health Impact Profile.

Dimension	Negative impact on OHRQoL *N* (%)	Items	Never *N* (%)	Hardly never/ Occasionally *N* (%)	Fairly often/ Very often *N* (%)
Functional limitation	13 (11.2)	Problem in pronouncing words	94 (81.0)	14 (12.1)	8 (6.9)
		Sense of taste worsened	101 (87.8)	7 (6.1)	7 (6.0)
Physical pain	11 (9.5)	Painful aching in mouth	89 (76.7)	21 (18.1)	6 (5.2)
		Uncomfortable to eat food	101 (87.0)	9 (7.8)	6 (5.2)
Psychological discomfort	10 (8.6)	Been self- conscious	93 (80.2)	15 (12.9)	8 (6.9)
		Felt tense or stressed	101 (87.1)	11 (9.5)	4 (3.4)
Physical disability	3 (2.6)	Diet unsatisfactory	103 (88.8)	10 (8.6)	3 (2.6)
		Interrupt meals	107 (92.2)	8 (6.9)	1 (0.9)
Psychological disability	8 (6.9)	Difficult relaxing	109 (94.0)	4 (3.4)	3 (2.6)
		Felt embarrassed	94 (81.7)	16 (13.9)	5 (4.3)
Social disability	0	Irritable with other people	112 (96.5)	4 (3.5)	0
		Difficulty doing your usual job	114 (98.3)	2 (1.7)	0
Handicap	7 (6.0)	Life less satisfying	103 (88.8)	7 (6.0)	6 (5.2)
		Unable to function	110 (94.8)	5 (4.3)	1 (0.9)

OHRQoL: Oral health-related quality of life.

The *N* in some cells is reduced because of missing values.

### Relationship between OHRQoL and associated factors

Table 4 shows bivariate associations between negative impact on OHRQoL and median OHIP-14 scores, and with background and general health variables.

**Table 4:** Background and general health variables associated with a negative impact on Oral health-related quality of life and median OHIP-14 scores.

Background variables	Negative impact on OHRQoL			Median OHIP-14 score		
	*N*	%	*p*	*N*	(IQR)	*p*
Sex			NS			NS
Female	11	18.0		60	0.5 (0–3)	
Male	13	23.6		54	2 (0–5)	
Age			NS			0.0055
65–74	8	34.8		23	3 (1–6)	
75–85	7	21.2		31	2 (0–5)	
> 85	9	15.0		60	0 (0–2)	
Living alone			NS			NS
Yes	19	20.9		89	1 (0–4)	
No	5	20.0		25	2 (0–4)	
Education			NS			NS
Basic	7	21.2		33	2 (0–3)	
Middle	13	25.5		50	1 (0–6)	
High	2	7.4		26	0 (0–2)	
Household income			0.009			NS
Low	14	33.3		41	2 (0–6)	
Middle	1	3.7		26	0.5 (0–2)	
High	5	24.0		21	2 (0–4)	
Number of medicines			NS			NS
0–4	7	25.0		9	5 (0–12)	
5–10	13	23.2		19	2 (0–3)	
> 10	3	11.5		80	1 (0–3.5)	
ADL score			0.020			NS
Score ≤ 2	10	35.7		28	2 (0–5.5)	
Score > 2	13	15.3		83	1 (0–3)	

ADL: Activity of Daily Living; OHRQoL: Oral health-related quality of life; OHIP: Oral Health Impact Profile; IQR; interquartile range.

Pearson’s Chi-squared and Fisher’s exact test, Kruskal-Walis, Mann-Whitney *U*-test, *p* < 0.05, NS: non-significant. IQR: interquartile range, The *N* in some cells is reduced because of missing values.

**Figure 2:**
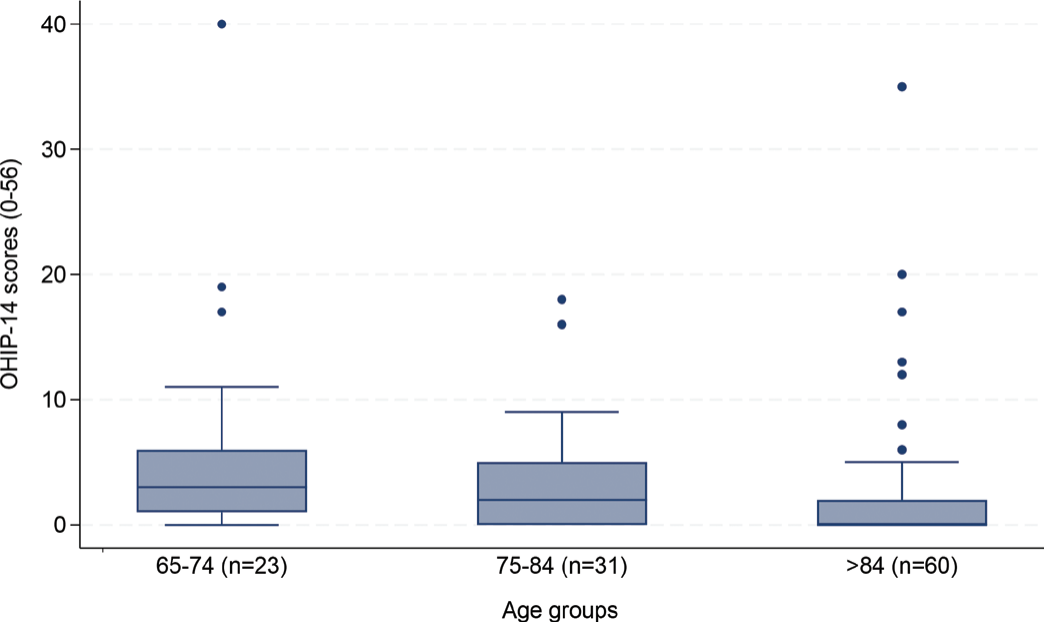
Boxplots illustrating the median with interquartile ranges (IQRs) of OHIP-14 total scores in participants within different age groups. *Kruskal-Wallis; p < 0.05. Dots in the figure represent outliers* (*N* = 114).

Younger individuals (65–74 years) had significantly higher median OHIP-14 scores compared with the other two age groups (Figure 2). Older adults who were less dependent on help (ADL score < 2) and those with lower income reported a negative oral health impact significantly more often than more dependent individuals and those with higher income, respectively.

Table 5 shows the results from bivariate analysis for associations between having a negative impact on OHRQoL and higher median OHIP-14 scores in relation to self-reported oral health status and symptoms. Individuals with a few remaining natural teeth and those who were edentulous had significantly higher median OHIP-14 scores. Having pain/discomfort and a bad taste or bad breath were significantly associated with a negative impact on OHRQoL and a higher median OHIP-14 score. Having loose teeth was significantly associated with a negative impact. Moreover, associations were found between poor self-perceived oral health status and having a negative impact and higher median OHIP-14 scores.

**Table 5:** Self-reported oral health variables associated with a negative impact on Oral health-related quality of life, and higher median OHIP-14 scores.

Self-reported oral health status and symptoms	Negative impact on OHRQoL			Median OHIP-14 sores		
	*N*	%	*p*	*N*	(IQR)	*p*
Number of teeth			NS			0.0099
Having all natural teeth	2	14.3		14	0 (0–1)	
Missing 1–4 teeth	5	11.4		44	0.5 (0–2)	
Missing 5–10	8	27.6		27	1 (0–6)	
Missing > 10	7	33.5		21	3 (1–5)	
Edentulous	2	25.0		8	3 (2–5)	
Tooth replacements						
Crown			NS			NS
Yes	9	15.5		58	1 (0–2)	
No	12	23.5		50	2 (0–4)	
Bridge			NS			0.0260
Yes	5	15.5		38	0 (0–2)	
No	18	26.9		65	2 (0–4)	
Tooth implant			NS			NS
Yes	1	12.5		8	1 (0–3)	
No	23	21.2		103	1 (0–4)	
Partial denture			0.010			0.0045
Yes	7	50.0		13	4 (2–12)	
No	17	17.0		99	1 (0–3)	
Full denture			0.043			0.0002
Yes	7	38.9		17	4 (2–9)	
No	17	17.7		95	0 (0–3)	
Oral symptoms last month						
Pain/discomfort			0.000			0.0000
Yes	11	57.9		18	9.5 (5–17)	
No	13	13.4		96	0 (0–2)	
Bleeding gingiva			NS			NS
Yes	5	29.4		16	2 (0–11.5)	
No	19	19.2		98	1 (0–3)	
Loose teeth			0.048			0.0552
Yes	5	45.5		11	7 (0–17)	
No	19	18.1		103	1 (0–3)	
Bad taste or bad breath			0.027			0.0162
Yes	6	46.2		13	5 (2–16)	
No	18	17.5		101	1 (0–3)	
Xerostomia			NS			NS
Yes	22	23.7		92	2 (0–4.5)	
No	1	5.0		20	0 (0–3)	
Self-perceived oral health			0.000			0.0001
Good	7	9.9		70	0 (0–2)	
Average	10	28.6		34	2 (1–6)	
Poor	7	70.0		10	10.5 (7–16)	

OHRQoL: Oral health-related quality of life; OHIP: Oral Health Impact Profile; IQR: interquartile range.

Results from the Pearson’s Chi-square test and Fisher’s exact test, Kruskal Wallis, Mann-Whitney *U*-test, with 95% CI < 0.05, NS: non-significant. The *N* in some cells is reduced because of missing values.

## Discussion

This study aimed to map self-reported oral health status, OHRQoL, and associated factors among older adults receiving HCS in Norway. It is one of few studies focusing on the self- reported oral health and OHRQoL of older adults ≥ 65 years living at home and depending on help from HCS. The findings indicate that a large proportion of care-dependent older adults retained their natural teeth, and crowns and bridges were common tooth replacements. Nearly one in five reported pain/discomfort during the last month and nearly half reported dry mouth symptoms. Despite this, the majority of care-dependent older adults rated their perceived oral health as good. Nearly half reported no impact on OHRQoL, while younger (< 75 years) and less dependent (ADL score < 2) individuals and those missing five or more natural teeth reported a negative impact on OHRQoL more often.

Around half of the participants in this study retained most of their natural teeth. This observation is in line with a previous study showing that older adults today retain more of their natural teeth [3, 4], and a large proportion in our study reported having tooth replacements such as crowns or bridges. Having fixed prosthetic appliances requires a good regular oral hygiene routine in order to maintain good oral health. However, 73% of all participants were highly dependent on daily care (ADL score > 2). Reduced self-care ability may affect individuals’ capability to maintain good oral hygiene, and thereby increase the risk of oral diseases such as caries and periodontitis [25].

The most common symptom was pain and discomfort, reported by almost one of five study participants. Moreover, a negative impact on the physical pain dimension was reported by 9.2%. Few studies have reported pain and discomfort among older adults, and measures were not standardized. Physical pain dimension in OHIP-14 has been reported more frequently (43%) among the general population of older adults in Norway [26]. Among dependent older adults, gum bleeding and pain were reported by 29% [27], and pain by 33.6% respectively [4]. Our findings indicate that the level of pain and discomfort is lower than previously reported. This may be due to a general improvement in oral health in the elderly. In addition, it may be speculated that medications used to relieve chronic pain conditions might further suppress oral symptoms [28, 29].

The high prevalence of xerostomia that was found among older adults in our study corresponds with results from previous studies on dependent older adults in Norway and other countries [27, 30, 31]. Nevertheless, the prevalence of xerostomia was higher in the present study than in a general population of young elderly individuals in Norway [32]. This may be attributed to the participants in our study being older adults with health problems, reliant on help from HCS. Prevalence of xerostomia has been shown to increase with age, medication use and reduced health conditions in populations aged 65–70 in Norway and Sweden [33]. The association between a number of medications used and xerostomia has been reported earlier [34]. However, in our study, and somewhat unexpectedly, we did not find any statistically significant associations between xerostomia and medication use, and the reason for this is not entirely clear. However, we had access only to information on the number of medicines, not specific classes or types of medication. However, medication use is just one of several factors associated with xerostomia in older adults. Other causes of xerostomia have been reported to be local factors such as poor dental health and oral infections, as well as chronic systemic or autoimmune diseases [35]. Prior research has established that dry mouth is one of the risk factors for dental caries. Therefore, the high prevalence of reported dry mouth symptoms is concerning [36].

Despite many participants indicating a negative impact on OHRQoL, 61.2% reported good self-perceived oral health status, a finding that is in line with previous studies [37, 38]. Holst et al. noted that 60% and 69% of individuals in the general population in Norway aged ≥ 60 years reported good self-perceived oral health status [39]. Quality of life has been described as ‘the individuals’ perception of their position in life in the context of the culture and value system in which they live and in relation to their goals expectations, standards and concern’ [40]. People assess quality of life based on their expectations and experiences, which can be influenced by factors such as their age or health condition. If they feel that their quality of life does not meet their expectations, they may perceive it as a poor quality of life [41]. Dahl et al. suggest that individuals who typically have good oral health may notice significant effects on their OHRQoL from relatively minor oral issues due to their high expectations regarding oral health [26].

More than half of the participants of our study reported experiencing one or more impacts of oral health status on daily life during the last year. In our study, age and daily function affected OHRQoL as individuals in the younger age group and with an ADL score < 2 more often reported poor OHRQoL compared with older individuals and those more dependent on help. This aligns with the results reported by Steele et al. who noted a lower impact of oral health problems on quality of life among individuals aged ≥ 70 years, than among younger individuals [42]. As suggested earlier, older and more dependent individuals may have lower expectations regarding their oral health than those who are younger and healthier. According to Baltes et al., individuals adjust their goals and expectations as they age and begin to prioritize different aspects of life. Consequently, they compensate for the losses and focus on what they now see as more important [43]. Previous results on OHRQoL among dependent older adults are not consistent. Despite different instruments have been used to investigate this, previous research indicates that OHRQoL may be challenging to capture among dependent older adults [44]. In Sweden, older adults who were more dependent on help reported having a lower quality of life [45], whereas elsewhere no relationship between ADL score and OHRQoL was found [46].

Several subjective oral health variables were associated with OHRQoL. Individuals with a higher number of natural teeth reported better OHRQoL more frequently, which accords with previous findings [30, 38, 45]. Having a full or partial denture was associated with poor OHRQoL, as found elsewhere [26, 38, 47]. Reduced chewing ability due to few teeth or poorly fitted dentures may impact QoL.

As the participants of this study were recruited by the HCS personnel using convenience sampling, the possibility of selection bias cannot be completely ruled out

Data collection was interrupted and delayed due to COVID-19 restrictions, which limited the number of participants. If data collection had proceeded as originally planned, a larger sample size could have been achieved.

However, data from Statistics Norway on 65+ year-olds show that the distribution of participants in the present study was similar in terms of sex, education level, and income compared with the general population [48]. Still, generalization of our findings should be done with caution, as users of HCS are a heterogeneous group in terms of general health and need for assistance.

Given the cross-sectional design of the present study, no conclusions on causal associations can be drawn. The age and health condition of the participants may impact the accuracy of reporting and filling out questionnaires, particularly in an interview-administered questionnaire, because the interviewer’s manner of asking questions may also influence participants’ responses [49]. Furthermore, recall bias cannot be excluded because participants’ cognitive and general health status may influence their responses to the questionnaire. Another factor that may have impacted the results is the lack of information regarding housing type, modernity, and ownership, which might have influenced participants’ ADL and well-being. Therefore, the findings of this study should be interpreted with caution.

Due to COVID-19 restrictions, clinical examinations could not be performed. Although our results are based on self-reported data, previous studies have demonstrated that self-reported oral health variables such as number of teeth and edentulousness are valid measures when conducting clinical examinations is not possible [50]. The use of validated questionnaires makes our results comparable with other studies.

Internal consistency of the OHIP-14 was assessed using Cronbach’s alpha and the calculated value was 0.85, which indicates high internal consistency of the questionnaire. OHIP-14 have been previously tested and found to be valid, for determining oral health-related impact on quality of life among older adults in Norway [51].

Because data on dependent elderly individuals are seldom available from population-based surveys, our findings from the present study shed light on the oral health conditions of a group with increasing treatment needs. The results from the present study provide important information about individuals in HCS who often have multiple health issues, including oral diseases. The WHO resolution on oral health from 2021 recommends an increased focus on a preventive and interprofessional approach in dental care to help older adults retain better oral health and OHRQoL [13]. The findings from this study can be used for decision support in planning dental and HCS for dependent older adults in the future.

## Conclusion

The results from this study indicate that half of older adults receiving assistance from HCS in Norway retain most of their natural teeth, few are edentulous, and xerostomia is common. Younger and less dependent individuals and those missing five or more natural teeth reported a negative impact on OHRQoL more often.

## Data Availability

The data that support the findings of this study are not publicly available because they contain information that could compromise the privacy of research participants. Further inquiries can be directed to the corresponding author (HIH).
